# Enhancement of long-horizon task planning via active and passive modification in large language models

**DOI:** 10.1038/s41598-025-91448-4

**Published:** 2025-02-28

**Authors:** Kazuki Hori, Kanata Suzuki, Tetsuya Ogata

**Affiliations:** 1 Faculty of Science and Engineering, Waseda University, Tokyo, Japan; 2 Artificial Intelligence Laboratories, Fujitsu Limited, Kawasaki, Kanagawa Japan; 3 Waseda Research Institute for Science and Engineering (WISE), Waseda University, Tokyo, Japan; 4 National Institute of Advanced Industrial Science and Technology, Tokyo, Japan

**Keywords:** Engineering, Mathematics and computing

## Abstract

This study proposes a method for generating complex and long-horizon off-line task plans using large language models (LLMs). Although several studies have been conducted in recent years on robot task planning using LLMs, the planning results tend to be simple, consisting of ten or fewer action commands, depending on the task. In the proposed method, the LLM actively collects missing information by asking questions, and the task plan is upgraded with one dialog example. One of the contributions of this study is a Q&A process in which ambiguity judgment is left to the LLM. By sequentially eliminating ambiguities contained in long-horizon tasks through dialogue, our method increases the amount of information included in movement plans. This study aims to further refine action plans obtained from active modification through dialogue by passive modification, and few studies have addressed these issues for long-horizon robot tasks. In our experiments, we define the number of items in the task planning as information for robot task execution, and we demonstrate the effectiveness of the proposed method through dialogue experiments using a cooking task as the subject. And as a result of the experiment, the amount of information could be increased by the proposed method.

## Introduction

In recent years, foundation and large language models (LLMs) have been actively applied in robotics^1–4^. Especially in robot task planning, LLMs can be used to divide complex natural language instructions into multiple lower-order tasks as input^5^, which has been difficult in past studies. Lower-order tasks are organized as lists or tables of short sentences, and they can be controlled by arbitrarily designing the prompts that are given to the LLM as input. However, previous studies^5,6^ have only applied LLMs to simple robotic tasks that consist of ten or fewer action commands, depending on the task, and have not considered long-horizon planning (issue I). This is because long-horizon tasks are more complex and zero-shot inference across multi-step tasks remains difficult. In addition, the ambiguity caused by polysemy and synonymy in natural language instructions and the lack of context-dependent information may inaccurately reflect the researcher’s intentions (issue II). The output can be tailored by elaborating natural language instructions in more detail, however, this design is expensive and especially difficult in long-horizon task planning.

On the other hand, the spread of inexpensive robot hardware and the development of imitation learning technology have made it possible to perform a variety of robot tasks^7–9^. Zhao et al.^7,8^ presented a low-cost fine-grained teleoperation system and the transformer-based method that learns various tasks. In the imitation learning framework, it can acquire a model directly from the robot experience if effective data for task execution are available. Our previous studies also showed that the imitation learning method can perform flexible object manipulations such as clothing^10,11^ buttoning^12^, in-air knotting^13^, and cooking scrambled eggs^14^. It is expected that the studies mentioned above will continue to develop and the skill set of robots will become even more enriched. In addition, research is being conducted to link simple language instruction with imitation learning^15–19^. In recent years, robot foundation models such as vision-language-action (VLA) models^20–22^ have been proposed, making it easier to link linguistic instructions with robot motions. Therefore, high-level motion planning that enables predictions using abstract task instructions is becoming increasingly important. The long-horizon task planning that we tackle in this study assumes that it is possible to execute robot skills from individual motion commands, and focus on refining abstract task planning using LLM.

To address issues I and II, this study utilized interactive prompt engineering to achieve longer and more complex action planning with a single dialogue example. The interactions with the collaborator would effectively refine the task planning. Here, the methods for interactively modifying the output of an LLM can be divided into two main categories: modifying the task plan based on sequential instructions from the collaborator (passive modification) and interactively modifying the task plan based on the uncertainty of the model output (active modification). In the former case, the collaborator points out incorrect parts of the task plan presented by the LLM, and enters prompts again to correct them. Although this can accurately reflect the collaborator’s intentions, it is very burdensome, because the task plan must be reviewed and instructions issued for every modification. Conversely, active modification allows the LLM to evaluate the uncertainty of its own planning output and interrogate the collaborator about the modifications. The cost of designing the initial natural language instructions is expected to be reduced since the LLM automatically presents the correction points, and the ambiguity of the task plan is resolved by answering the question.

We have attempted to combine both modification methods to advance task planning while reducing the number of concrete examples given as LLM prompts. The proposed method constructs a prompt such that the LLM side requests missing information in response to a goal described using language instructions, and information is collected interactively^23^. In addition, the human side provides the missing information using only text or text with images, and the final task planning result is output. Few have researched the active modification of task plans by LLMs, and the proposal of the method combined with passive modification is an important contribution of this study.

The remainder of this paper is organized as follows. Section 2 briefly describes previous research on robot task planning using LLMs and its challenges. Section 3 describes the task-plan generation process for LLMs, including active and passive modification, and organizes the constructed prompts. Sections 4 and 5 present the setup and results of dialog experiments on robot task planning using ChatGPT^24^, a type of interactive LLM. Finally, Section 6 provides the conclusions drawn from the study findings and future prospects.

## Related works

### Offline task planning using an LLM

Offline task planning is important in long-horizon robot tasks. The more complex the task, the more important it is to have clear planning before taking action^12^. Many studies have investigated offline robot task planning using LLMs to convert human task instructions into multiple action plans. The action plans may be output in table^25^ and code^26,27^ formats. The most well-known action planning models are SayCan^5^ and RT-1^28^, and the latter model includes action execution . RT-1 uses an LLM to evaluate the probability of selecting each skill to determine the next action. In addition, research has been conducted to apply multimodal information to offline task planning. Palo et al.^29^ incorporated a vision language model (VLM) into task planning and proposed an efficient reinforcement learning method including subgoal generation. Zeng et al.^30^ further proposed a task planning method that combines an LLM, VLM, and audio language model (ALM) and demonstrated that this system improved zero-shot inference capabilities. In addition, Wu et al.^31^ performed real robot tasks with flexible planning by inputting robot state and image information into an LLM.

By contrast , long-horizon task planning remains difficult to achieve. Several recent studies have combined symbolic motion planners with LLMs to handle long-horizon robot tasks. Liu et al.^32^ presented a method to improve the feasibility of task prediction by LLMs using a scene graph of the environment. By using a structured representation of the scene graph, this method can better predict long-horizon tasks compared to other methods that combine symbolic planners. However, many studies lack generality because the skills that are considered are pre-defined. Motor commands that are not predefined reduce the quality of predicted task plans due to the ambiguity inherent in natural language. Studies on behavior tree generation have also been conducted using step-by-step planning^33^, and long-horizon task planning functions have been strengthened by incorporating a classical planner^34^; however, these studies require multiple examples to be incorporated into the prompt. By contrast, our study interactively refines the planning process in advance such that creating multiple examples is unnecessary.

### Online task planning with feedback

To achieve task planning in dynamic environments, approaches that refine predicted task plans have been investigated. The mainstream approach in this field is to create feedback based on image information. Song et al.^35^ and Yang et al.^36^ used visual questioning and answering (VQA) to determine the success or failure of task execution from an image and revised the task plan accordingly. As the performance of the foundation model improves, situational understanding using VQA enables flexible task evaluation via prompt engineering. Several studies have also been conducted to improve the accuracy of task replanning by generating failure explanations^37–39^. However, the accuracy of plan revision depends on the quality of the feedback (recognition accuracy based on image information) when using these methods.

Efforts have also been made to reduce the error between the LLM output and the real environment through interaction with external knowledge. Vemprala et al.^27^ used an interactive LLM to generate robot motion codes at the programming level and adjust these codes by providing feedback using a simulator. Ding et al.^40^ and Zhao et al.^41^ improved the accuracy of planning by collecting task information using the commonsense provided by an LLM. Another method using the dialogue history between multiple agents has also been proposed^42^. In addition, Lin et al.^43^ contributed to reducing the cost of plan revision by providing higher-order planning that receives feedback based on dual process theory. Using environmental information linked to linguistic information is also effective in improving the accuracy of planning. Rena et al.^44^ proposed a method that uses LLMs to infer scene graphs, generate plans across large environments, and employ iterative replanning to ensure that scene graph constraints are not violated. Chen et al.^45^ created an NLMap that combines detection queries and maps and generates action candidates by matching them against queries output by an LLM.

Among feedback methods, dialogue-based information gathering^6,46,47^ is costly but highly accurate, and it is effective for plan revision. Huang et al.^47^ incorporated closed-loop linguistic feedback based on action success signals into task planning in addition to image feedback, which includes the detection of successes/failures and scene descriptions. Ren et al.^6^ studied the use of active modification in online sequential task planning. They attempted to clarify action plans by evaluating the uncertainty of task plans based on natural language instructions from humans and visual information from the robot, and by responding to questions accordingly. The ability to actively gather information is crucial for the task execution of real robots^48,49^, and this is a focus of the present study.

One of the contributions of this study is a Q&A process in which ambiguity judgment is left to LLM. Planning that includes environmental information such as images and scene graphs from the beginning of planning increases preparation costs. Furthermore, it is difficult to include all the information necessary for long-horizon tasks in environmental information. By sequentially eliminating ambiguities contained in long-horizon tasks through dialogue, our method increases the amount of information included in movement plans. Modifying the task planning interactively is labor-intensive work, however, active modification by Q&A is considered to have advantages in terms of the scope of application because it is written in natural language. Thus, active modification is expected to contribute to reducing the design cost of natural language instructions. This study aims to further refine action plans obtained from active modification through dialogue by passive modification, and few studies have addressed these issues for long-horizon robot tasks. The contributions of this study are summarized as follows:

- We propose an active modification method for task planning through a question-and-answer process based on ambiguity judgment using an LLM.
- We evaluate the effect of combining active and passive modification methods using visual information.
- We evaluate the proposed method through experiments using one concrete example of a dialogue input.

## Method

**Fig. 1:**
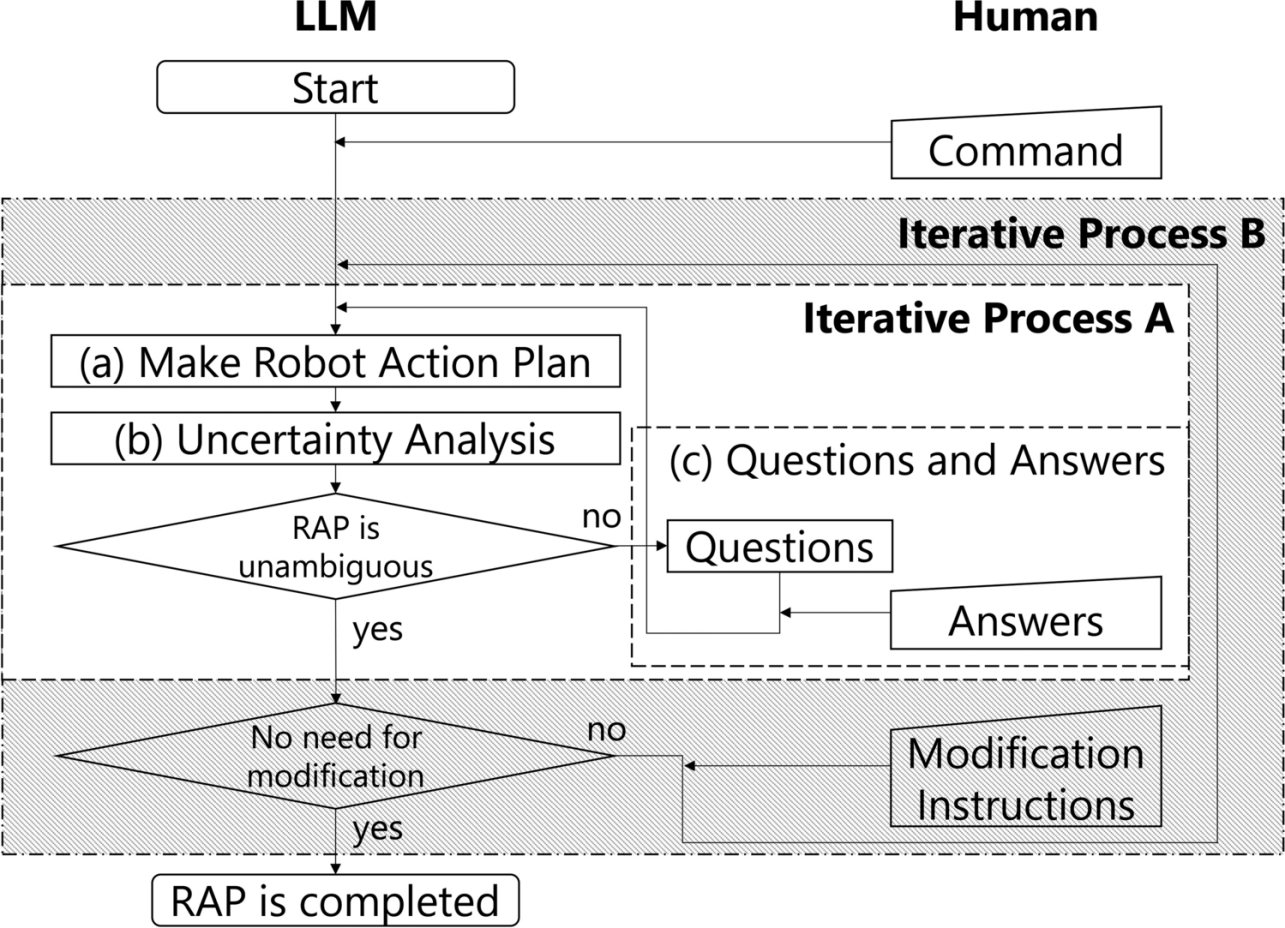
Process overview of the proposed method.

This study presents a method for generating a task plan for a robot through a dialog between a human and an LLM. An overview of the proposed method’s processing procedure is shown in Fig. 1. The proposed method consists of two iterative processes, Process A and Process B, each of which plays the role of active and passive modification. Since process B incorporates process A, the entire process sequentially undergoes multiple iterations. In each process, the entire dialog history is included in a prompt. Thereby, after a natural language instruction (Command), which simply conveys the task instruction, is passed from the human to the LLM, and the LLM interactively modifies the output toward the described goal. The details of each process are described below.

The proposed method is completed within the dialogue with the LLM, and all behaviors of the method are controlled by prompts. Therefore, by combining the simple prompts described in this section, it is possible to improve the robot task plans that are output by the LLM.

### Active modification

We explain the active modification performed in Process A. Active modification requires the LLM to evaluate the uncertainty of its own planning output and to pose questions to the collaborator about the modifications. The LLM is expected to reduce the cost of designing the initial natural language instructions because it automatically suggests modifications and the collaborator answers questions to resolve ambiguities included in the task planning results. The active modification process comprises the following three steps.

- Formulate the Robot Action Plan
- Uncertainty Analysis
- Questions and Answers

For each process, the appropriate prompts are designed to produce the desired LLM output. The following sections detail each step.

#### Make robot action plan

First, the robot’s task plan is output from the natural language instructions given by the human to the LLM. The output planning is summarized and output in tabular format. In this paper, the task plan is named the Robot Action Plan (RAP). Below is an example of a prompt that implements step (a) and a sequence of actions output by the LLM.
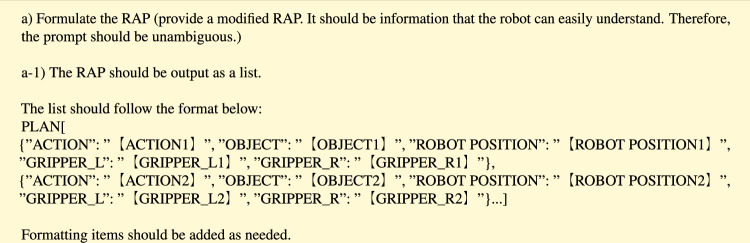


The RAP is generated by the LLM based on the Command first given to the LLM and the information obtained from the questions and answers described below. However, the first process is generated using only the Command information. The RAP consists of multiple action sequences, each of which consists of the following five columns; ACTION” indicates the action category, “OBJECT” indicates the action target, “ROBOT POSITION” indicates the robot’s position, “GRIPPER_L” indicates what the left arm is grasping, and “GRIPPER_R” indicates what the right arm is grasping. It is expected that the comprehensive and structured representation described above will facilitate robot control at a later stage.

The category class in the RAP in this experiment was designed to perform a minimum of pick-place tasks and move tasks. Since the proposed method only verified the effectiveness of combining passive and active modifications, there is still room for improvement in the format of prompts and RAP. For example, a passive change process may be more efficient in a GUI format.

However, there may sometimes be difficulty in expressing a sequence of actions with only the above items in complex robot tasks. Therefore, in this study, we recommended that LLMs insert additional RAP columns at their own discretion by including the statement “It is recommended to add formatting items as needed.” This allows for the flexible modification of task plans through dialog with humans.

#### Uncertainty analysis

In the next step, the LLM analyzes the ambiguities in the generated RAP and outputs the analysis results as sentences. By analyzing the ambiguity, the LLM determines whether the current RAP is sufficient to execute the robot’s action. This is based on the “step-by-step” concept^50^, which is useful in prompt engineering, and increases the accuracy of the final LLM output. In this study, ambiguity is considered as uncertainty in prediction, and its analysis is based on the subjectivity of the LLM. While some previous studies have taken the approach of outputting the probability as a numerical value to the LLM^6^, in this study, the LLM determines whether or not to include ambiguity internally. This is accomplished by the following statements in the prompts.
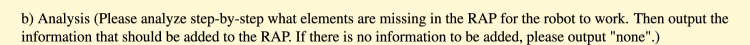


As described in the previous subsection, the RAP is in table notation, which allows item-by-item analysis and streamlines the ambiguity analysis. Although the LLM in the proposed method makes a binary decision about whether there are ambiguities in the output based only on the prompt, previous experimental results showed that our method can also make it with a certain degree of certainty^23^. In this process, if the LLM determines that the RAP is unambiguous, iterative process A is terminated, and the output RAP is handed over to process B (passive modification).

#### Questions and answers

If the “Uncertainty Analysis” determines that there is an ambiguity in the RAP, the LLM and human interact to gather additional information for disambiguation. In this process, the LLM first generates questions to disambiguate the RAP. These questions are generated based on the results of the “Uncertainty Analysis.” By inputting human responses to the above questions, the LLM side incorporates the responses in the task planning. Since the answers are provided by humans, the information added to the RAP is, to some extent, guaranteed to be relevant to the task. This process is realized by the following description in the prompt.
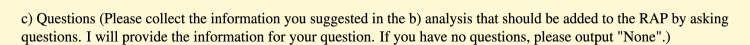


In the proposed method, multiple questions may be output simultaneously, and the collaborator may similarly input multiple answers. The collaborator may refuse to answer questions for which he/she has no response. This reduces the burden on the collaborator and prevents unimportant information from being added to the RAP. The information obtained here is also used in the process (b) after the iterations. Steps (a–c) are repeated until the RAP in Process A is completed.

### Passive modification

In the passive modification process, the collaborator points out the incorrect parts of the RAP output by the active modification process and re-enters prompts to modify the RAP. This compensates for those RAP items that the LLM cannot perform. There are various types of passive modifications, such as addition, deletion, or replacement of information. Any of these modifications is possible, depending on the design of the human instructions. The above three items were verified experimentally.

This study applied passive modification to a robot task plan that has undergone active modification through iterative process B in Fig. 1. After iterative process A, if there is a modification instruction by the collaborator, iterative process A is performed again with the modification instruction. In this study, the basic timing for giving modification instructions is after iterative process A. However, experiments have confirmed that modification instructions can also be accepted at the timing of Answers in (c) Questions and Answers in iterative process A.

## Experimental setup

### Dialog task for task planning

In the proposed method, it is difficult to quantitatively evaluate whether the amount of information added through dialogue benefits the robot task plan. Therefore, we discuss the effectiveness of the proposed method by considering specific dialogue-planning results. Dialog experiments 1–3 were conducted to verify the effectiveness of the proposed method for action planning in a long-horizon task. A robot cooking task was selected as the long-horizon task for verification. The cooking task is widely required for general-purpose household robots and has been widely studied^14,51,52^. In this experiment, the subjects were asked to cook scrambled eggs (Task 1) and cut carrots (Task 2). These tasks were selected based on the experimental setup of the previous study^23^. Here, the commands given to the LLM are “Make scrambled eggs.” and “Cut carrots.”. The above commands were designed to be simple and to include significant ambiguity about the cooking process and environment. This experiment investigated whether this ambiguity could be resolved using active and passive modification.

In Experiments 1 and 2, we used ChatGPT version gpt-4-0613 as the LLM^24^. In Experiment 3, we used ChatGPT version gpt-4-vision-preview as the Vision Language Model. We used 2253 tokens in the prompts for the initial instructions, including the concrete example. The temperature was set to 0 in all experiments except for a portion of Experiment 1.

The following three experiments were conducted for planning the above robot task.

**Fig. 2:**
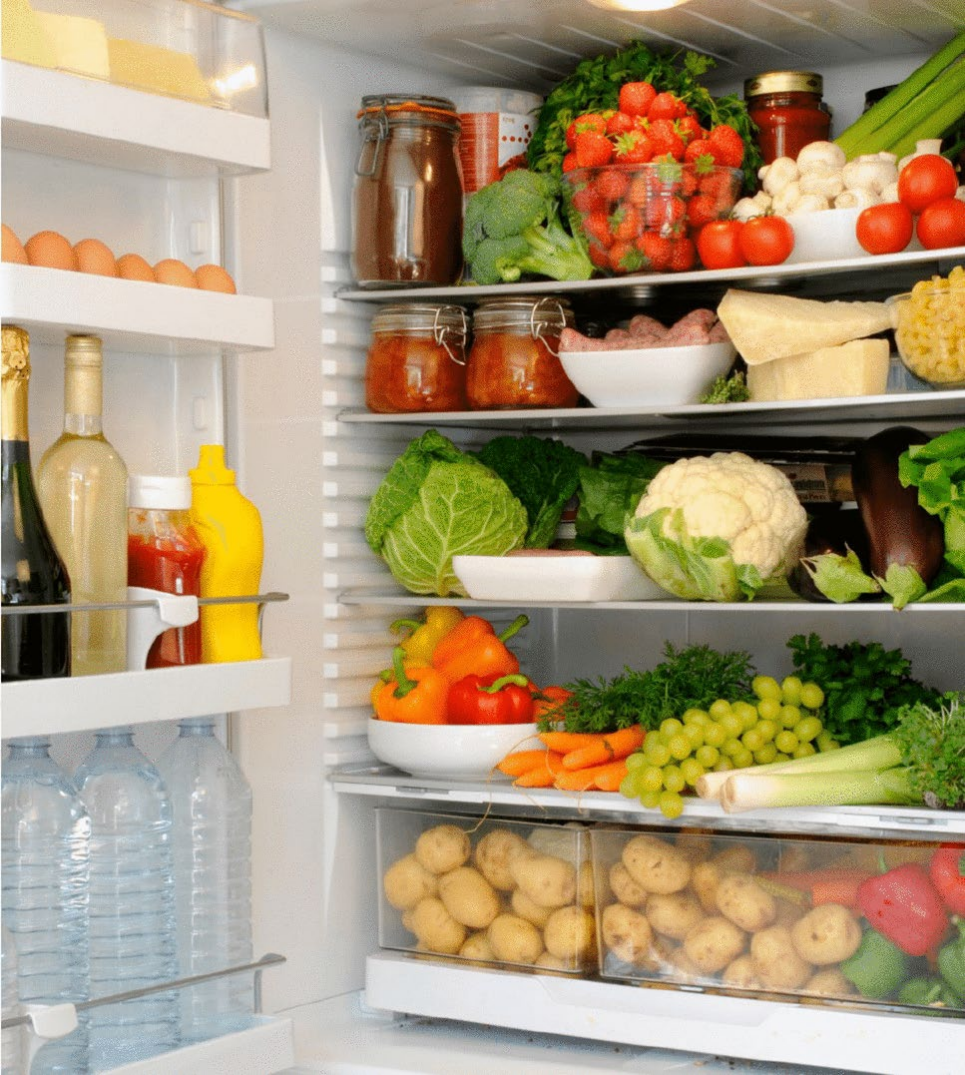
Input images in Task 1 (Addition of location information) of passive modification using visual information in Experiment 3.

**Fig. 3:**
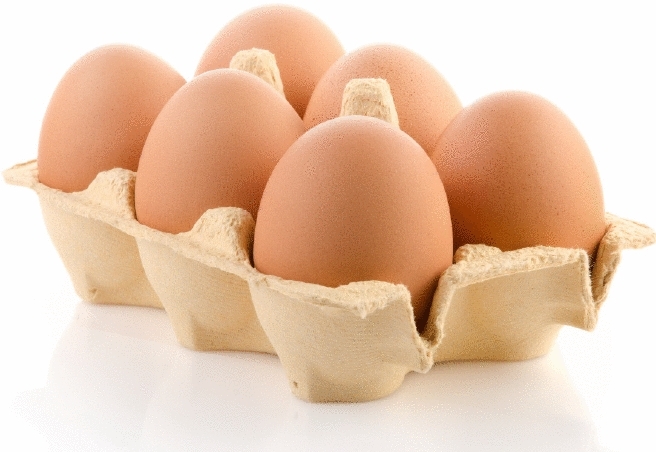
Input images in Task 2 (Addition of feature information) of passive modification using visual information in Experiment 3.

- Experiment 1: Comparison of RAP before and after active modification We confirmed the effectiveness of active modification in long-horizon tasks and active modification in offline task planning. By comparing the RAP generated by the initial step (a), which did not involve an iterative process, with the RAP generated by the final processing, to establish whether active modification improved the RAP. To quantify how much the amount of information in the RAP increased before and after active modification was applied, we also compared the amount of information in the RAP before and after active modification. As experimental data for comparison, we conducted 10 trials of a scrambled egg cooking task. Using these data, we obtained the displacement of the amount of information before and after passive modification. In addition, to investigate the effect of temperature change on the RAP formation, the temperature of the LLM was set to 0 or 1, respectively. Using this data, we also determined the average number of words used per response. This would be a simple indication of the ease of answering the questions in the output.
- Experiment 2: Validation of passive modification The following three passive modifications were applied to the RAP generated by the active modification in Experiment 1 to confirm that the active and passive modifications can be flexibly used together.
  1. Addition of information (Modification instructions: Add the process of seasoning with ketchup.)
  2. Deletion of information (Modification instructions: Do not use ketchup after all.)
  3. Replacement of information (Modification instructions: Use butter, not oil, after all.)
  Only the cooking of scrambled eggs (Task 1) was used as the validation task. In addition, by passive modification, we attempted to compensate for the missing information in the RAP generated by active modification. Through this experiment, we confirmed the effectiveness of combining active and passive modification in a long-horizon task.
- Experiment 3: Passive modification using visual information (addition of location and feature information) We confirmed that the interactivity of the RAP modification method can be variously extended by utilizing visual information. One of the targets of this paper is to verify the performance of high-level planning using LLM for imitation learning. This experiment assumes a case where the image is used to modify the action plan when it is difficult to execute the action by motion learning model. To this end, we performed the following two tasks as passive modifications using images.
  1. Addition of location information (Modification instructions: Add information on the location of the egg based on the image.)
  2. Addition of feature information (Modification instructions: Please add information based on the picture about the appearance of the egg.)
  The input images for each task are Figs. 2 and  3. Passive modifications were applied to the RAPs after active modifications were applied by inputting these with the modification instructions. The validation task was cooking scrambled eggs.

### Prompt

The prompts provided to the LLM in the proposed method are described below. The overall prompt consists of the following five components.

- Role: Describe the LLM’s role and purpose
- Prerequisites: Describe the robot’s capabilities and prerequisites such as its initial position
- Process: Describes the process of active modification
- Output format: Describes the format of the output
- Example: Describe two types of specific examples

“Role” and “Prerequisites” described only conditions that related to the robot’s capabilities and the initial state of the experiment (e.g., position). In this paper, we assume a mobile manipulator with a dual-arm gripper and write the prompt. Information such as sensors such as tactile sensors or cameras are not listed. This is because the proposed method aims to output a general long-horizon task plan from ambiguous action instructions. It may be possible to make the plan more specific by recording environmental information such as various sensor information and scene graphs in detail, but it will be costly to prepare. Specifically, the following conditions were described in conjunction with “Prerequisites” and “Role.”
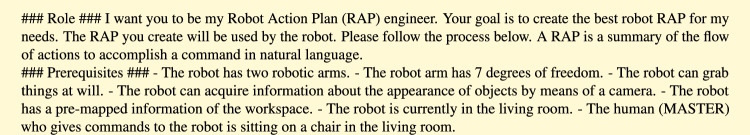


The “Process” and “Output format” fields are populated through the active modification process described in the previous section. The process for passive modification is not described in the prompt.

In the “Example” part, we enter dialogs for creating the RAP in active modification. In this study, only one dialogue example is used. As a concrete example of input/output, the RAP for the task “Get an energy drink from the refrigerator in the kitchen” is input. The dialog is written as below.
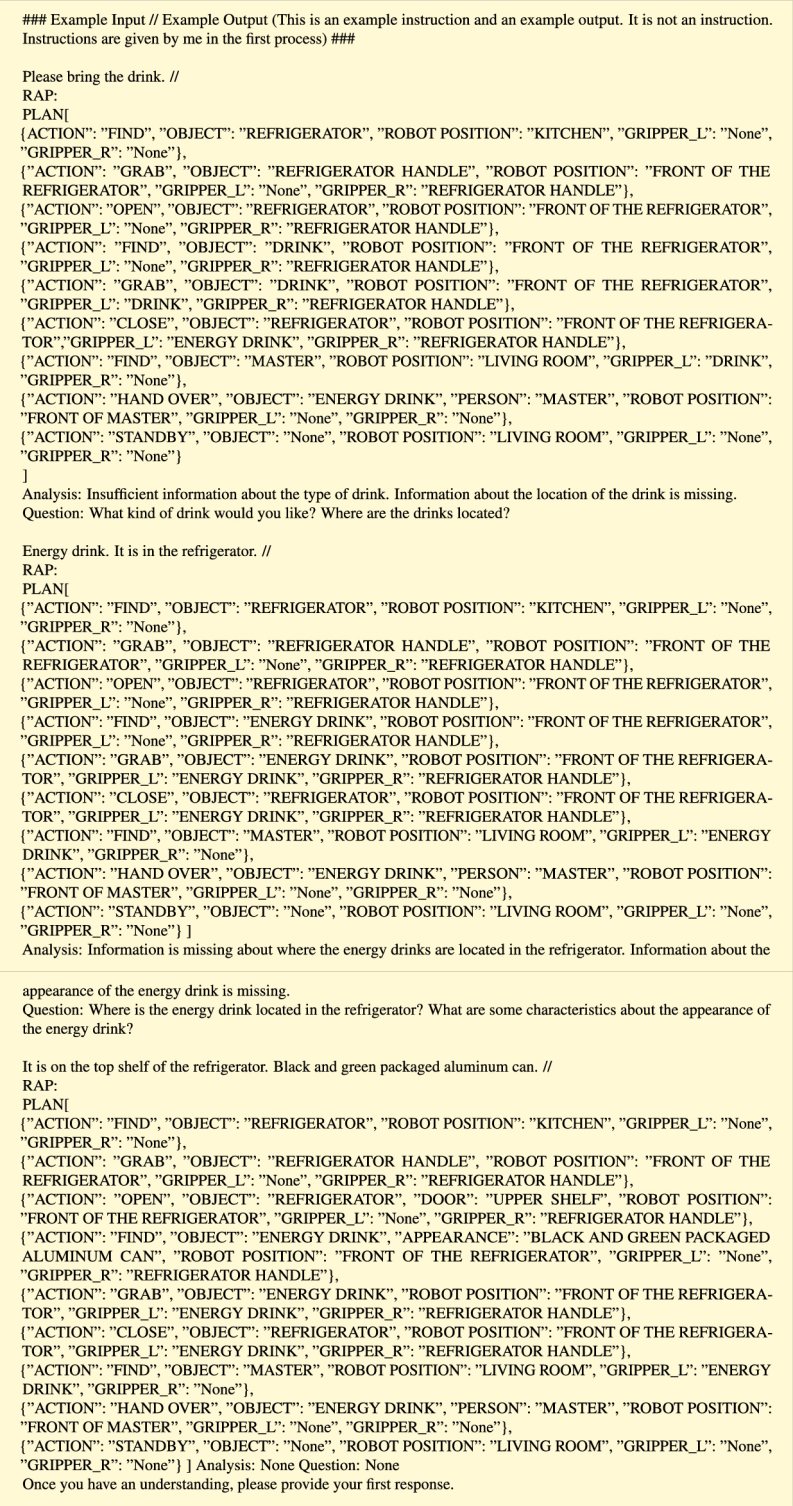


The Role to Example prompt above is always entered when making a request to the LLM during an experiment. Also, the initial conversation that communicates the command to the LLM is always entered. However, the RAP, Analysis, and Question output by the LLM are only entered for the most recent output. This is done to reduce the number of tokens used.

## Results and discussion

### Experiment 1

**Fig. 4:**
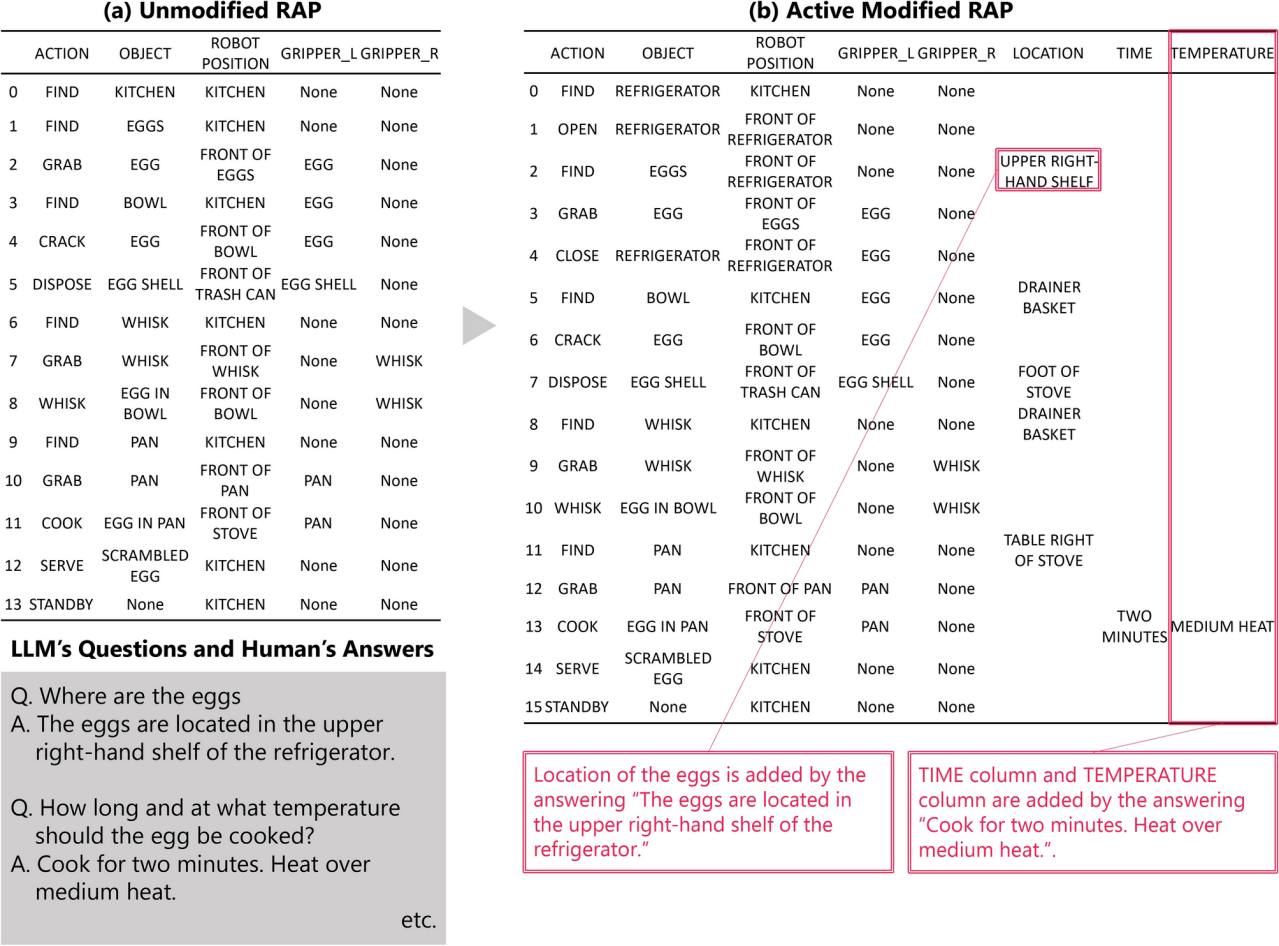
Comparison of RAPs before and after active modification in Task 1 (Make scrambled eggs.) in Experiment 1.

First, we verified the effectiveness of active modification. The results of the dialog experiment of Task 1 (Command: Make scrambled eggs.) are shown in Fig. 4. Tables (a) and (b) in the figure summarize the RAPs before and after active modification. The LLM determined that Table (b) was sufficiently clear after its question-and-answer interaction with the collaborator (shown in the lower left of the figure). Comparing tables, the RAP clearly reflects the information obtained from the question-and-answer interaction. The questions from the LLMs were related to the position of the eggs, the heating time, and the temperature of the heat. The response to these answers confirmed that location of the eggs was added to the RAP by the collaborator’s response “The eggs are located in the upper right-hand shelf of the refrigerator.”. It was also confirmed that TIME column and TEMPERATURE column were added to the RAP by the collaborator’s response “Cook for two minutes. Heat over medium heat.”. Note that the LLM’s question and the collaborator’s answers shown in the lower left-hand corner are a single example, and several other questions and answers were posed and addressed through active modification.

**Fig. 5:**
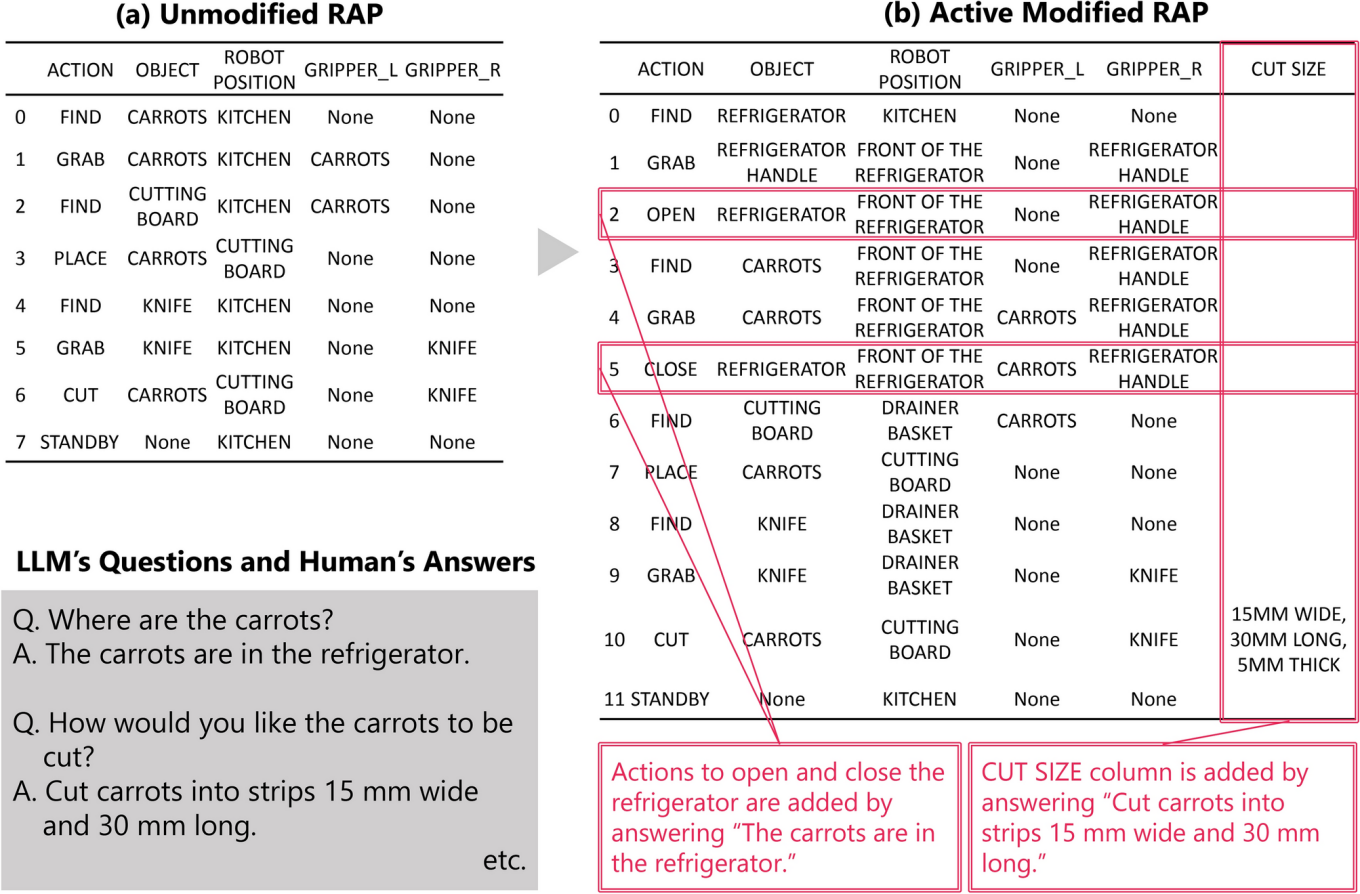
Comparison of RAPs before and after active modification in Task 2 (Cut carrots.) in Experiment 1.

Next, the results of the dialogue experiment of Task 2 (Command: Cut carrots.) are shown in Fig. 5. Each table summarizes the RAPs before and after active modification. Similar to the results of Task 1, the RAPs in Table (b) reflect the various information obtained from the question-and-answer session with the collaborator. For example, the collaborator’s response “The carrots are in the refrigerator” added actions to open and close the refrigerator to the RAP. Additionally, the collaborator’s response “Cut carrots into strips 15 mm wide and 30 mm long” added the SIZE column to the RAP. The results of Task 1 and Task 2, evidence that the proposed method can reflect the responses of the collaborators and clarify the task plan through dialogue. Both Task1 and Task 2 indicate that the action plan can be extended to the pre-defined RAP output format. This ability to add items is important when planning more complex tasks that are difficult to define in advance.

**Fig. 6:**
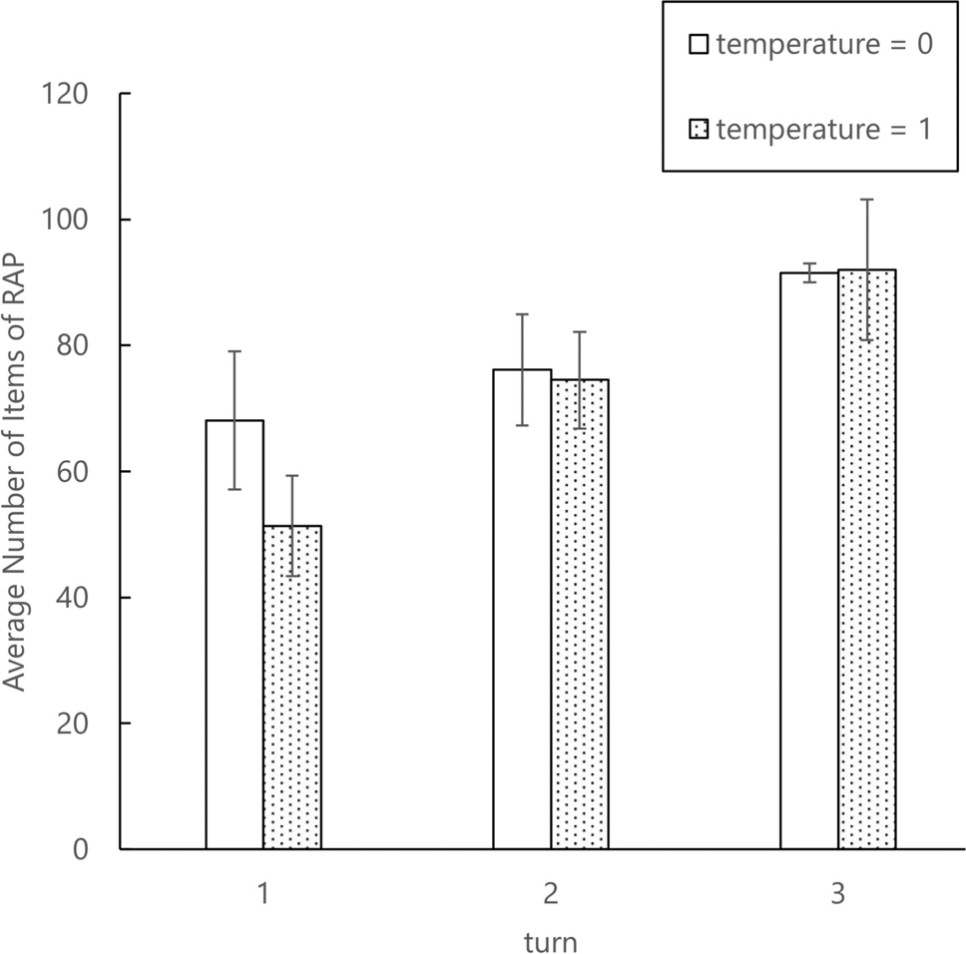
In Task 1 (Make scrambled egg.) of Experiment 1, the change in the number of RAP items before and after the active modification. The error bars represent the standard deviation (n=10).

We quantitatively evaluated the effectiveness of the proposed method. Figure 6 shows the change in the number of RAP items for Task 1 (Make scrambled egg.) in Experiment 1, before and after applying active modification. Error bars indicate standard deviation. The number of RAP items increases with the number of turns. Specifically, when the temperature of the LLM was set to 0, the number of RAP items increased by an average of 53% before and after the active modification. In addition, with temperature=0, the standard deviation decreases with each turn, but with temperature=1, it remains large. Average number of words used per response was 6.7. This indicates that the LLM outputs questions that can be easily answered in about 7 words. In the above analysis, we defined the number of items in the RAP as information required for task planning. The problem with this definition is that it equally considers necessary and unnecessary information when unnecessary information is described in the RAP. Appropriately defining the amount of information in the RAP will be investigated in future research.

Although the change in the number of items we adopted in this analysis may not be suitable for evaluating the accuracy of the task planning, we think it demonstrated that the amount of information has increased. So, the original purpose of this paper to reduce ambiguity has been evaluated. The proposed method assumes that there is a robot motion model based on imitation learning at the subsequent stage, and that unnecessary information will be removed if feedback from the motion prediction model is returned^53,54^. Although we did not measure the task planning time in this experiment, we confirmed that it would not take an experienced robot user that much time. Also, we did not see any categories output that were significantly different than expected, since each category class is defined by dialog examples provided in the prompt.

### Experiment 2

Next, we verified the effectiveness of passive modification in the proposed method. Table 1 shows the RAP before passive modification in Experiment 2. This is the RAP after active modification in Task 1 of Experiment 1. Tables 2 through 5 show the results of applying various passive modifications to the RAPs in Table 1.

**Table 1:** RAP before passive output modification in Experiment 2.

	ACTION	OBJECT	ROBOT POSITION	GRIPPER_L	GRIPPER_R	HEAT	DURATION
0	FIND	REFRIGERATOR	KITCHEN	None	None		
1	GRAB	REFRIGERATOR HANDLE	FRONT OF THE REFRIGERATOR	None	REFRIGERATOR HANDLE		
2	OPEN	REFRIGERATOR	FRONT OF THE REFRIGERATOR	None	REFRIGERATOR HANDLE		
3	FIND	EGGS	FRONT OF THE REFRIGERATOR	None	None		
4	GRAB	EGGS	FRONT OF THE REFRIGERATOR	EGGS	None		
5	CLOSE	REFRIGERATOR	FRONT OF THE REFRIGERATOR	EGGS	None		
6	FIND	OIL	PANTRY	EGGS	None		
7	GRAB	OIL	PANTRY	EGGS	OIL		
8	PLACE	EGGS	KITCHEN COUNTER	None	OIL		
9	FIND	BOWL	CABINET	None	OIL		
10	GRAB	BOWL	CABINET	BOWL	OIL		
11	PLACE	BOWL	KITCHEN COUNTER	None	OIL		
12	POUR	OIL	STOVE	None	None		
13	GRAB	EGGS	KITCHEN COUNTER	EGGS	None		
14	CRACK	EGGS	OVER BOWL	None	None		
15	DISCARD	EGG SHELLS	TRASH CAN	None	None		
16	FIND	SALT	PANTRY	None	None		
17	GRAB	SALT	PANTRY	SALT	None		
18	SEASON	EGGS IN BOWL	KITCHEN COUNTER	SALT	None		
19	FIND	PEPPER	PANTRY	None	None		
20	GRAB	PEPPER	PANTRY	PEPPER	None		
21	SEASON	EGGS IN BOWL	KITCHEN COUNTER	PEPPER	None		
22	FIND	WHISK	DRAINER BASKET	None	None		
23	GRAB	WHISK	DRAINER BASKET	WHISK	None		
24	BEAT	EGGS IN BOWL	KITCHEN COUNTER	WHISK	None		
25	FIND	PAN	RIGHT OF STOVE	None	None		
26	GRAB	PAN	RIGHT OF STOVE	PAN	None		
27	PLACE	PAN	STOVE	None	None		
28	POUR	BEATEN EGGS	STOVE	None	None		
29	COOK	EGGS IN PAN	STOVE	None	None	MEDIUM	2 MINUTES
30	FIND	SPATULA	DRAINER BASKET	None	None		
31	GRAB	SPATULA	DRAINER BASKET	SPATULA	None		
32	STIR	EGGS IN PAN	STOVE	SPATULA	None		
33	SERVE	SCRAMBLED EGGS	KITCHEN COUNTER	None	None		
34	STANDBY	None	LIVING ROOM	None	None		

Addition of information

- Table 2 is the result of passive modification to Table 1 with the modification instruction “Add the process of seasoning with ketchup.” A comparison of Table 1 and Table 2 shows that the information in the italic part of Table 2 has been added by passive modification. They are the process of seasoning with ketchup, which indicates that passive modification has been applied to add information as per the modification instructions.
- Table 3 shows the RAP modified by iterative process A (active modification) after the output of Table 2. One question asked in the iterative process was “Where is the ketchup located and how much should be used for seasoning?” This question was asked to resolve the ambiguity inherent in the modification instructions to add the process of seasoning with ketchup. The responses of the collaborator to this question were “The ketchup is in the refrigerator.” and “Put about 10g of ketchup.” Through this question and response, the information shown in the italic part of Table 3 has been added. This information is about the location of the ketchup and the amount of ketchup. This result indicates that the interaction of iterative processes A and B in the proposed method works correctly and refines the RAP.

**Table 2:** RAP after passive modification in Task 1 (Addition of information) in Experiment 2.

	ACTION	OBJECT	ROBOT POSITION	GRIPPER_L	GRIPPER_R	HEAT	DURATION
0	FIND	REFRIGERATOR	KITCHEN	None	None		
1	GRAB	REFRIGERATOR HANDLE	FRONT OF THE REFRIGERATOR	None	REFRIGERATOR HANDLE		
2	OPEN	REFRIGERATOR	FRONT OF THE REFRIGERATOR	None	REFRIGERATOR HANDLE		
3	FIND	EGGS	FRONT OF THE REFRIGERATOR	None	None		
4	GRAB	EGGS	FRONT OF THE REFRIGERATOR	EGGS	None		
5	CLOSE	REFRIGERATOR	FRONT OF THE REFRIGERATOR	EGGS	None		
6	FIND	OIL	PANTRY	EGGS	None		
7	GRAB	OIL	PANTRY	EGGS	OIL		
8	PLACE	EGGS	KITCHEN COUNTER	None	OIL		
9	FIND	BOWL	CABINET	None	OIL		
10	GRAB	BOWL	CABINET	BOWL	OIL		
11	PLACE	BOWL	KITCHEN COUNTER	None	OIL		
12	POUR	OIL	STOVE	None	None		
13	GRAB	EGGS	KITCHEN COUNTER	EGGS	None		
14	CRACK	EGGS	OVER BOWL	None	None		
15	DISCARD	EGG SHELLS	TRASH CAN	None	None		
16	FIND	SALT	PANTRY	None	None		
17	GRAB	SALT	PANTRY	SALT	None		
18	SEASON	EGGS IN BOWL	KITCHEN COUNTER	SALT	None		
19	FIND	PEPPER	PANTRY	None	None		
20	GRAB	PEPPER	PANTRY	PEPPER	None		
21	SEASON	EGGS IN BOWL	KITCHEN COUNTER	PEPPER	None		
*22*	*FIND*	*KETCHUP*	*None*	*None*	*None*		
*23*	*GRAB*	*KETCHUP*	*None*	*KETCHUP*	*None*		
*24*	*SEASON*	*EGGS IN BOWL*	*KITCHEN COUNTER*	*KETCHUP*	*None*		
25	FIND	WHISK	DRAINER BASKET	None	None		
26	GRAB	WHISK	DRAINER BASKET	WHISK	None		
27	BEAT	EGGS IN BOWL	KITCHEN COUNTER	WHISK	None		
28	FIND	PAN	RIGHT OF STOVE	None	None		
29	GRAB	PAN	RIGHT OF STOVE	PAN	None		
30	PLACE	PAN	STOVE	None	None		
31	POUR	BEATEN EGGS	STOVE	None	None		
32	COOK	EGGS IN PAN	STOVE	None	None	MEDIUM	2 MINUTES
33	FIND	SPATULA	DRAINER BASKET	None	None		
34	GRAB	SPATULA	DRAINER BASKET	SPATULA	None		
35	STIR	EGGS IN PAN	STOVE	SPATULA	None		
36	SERVE	SCRAMBLED EGGS	KITCHEN COUNTER	None	None		
37	STANDBY	None	LIVING ROOM	None	None		

The Italic are the added information.

**Table 3:** RAP after active modification in Task 1 (Addition of information) in Experiment 2.

	ACTION	OBJECT	ROBOT POSITION	GRIPPER_L	GRIPPER_R	*AMOUNT*	HEAT	DURATION
0	FIND	REFRIGERATOR	KITCHEN	None	None			
1	GRAB	REFRIGERATOR HANDLE	FRONT OF THE REFRIGERATOR	None	REFRIGERATOR HANDLE			
2	OPEN	REFRIGERATOR	FRONT OF THE REFRIGERATOR	None	REFRIGERATOR HANDLE			
3	FIND	EGGS	FRONT OF THE REFRIGERATOR	None	None			
4	GRAB	EGGS	FRONT OF THE REFRIGERATOR	EGGS	None			
5	CLOSE	REFRIGERATOR	FRONT OF THE REFRIGERATOR	EGGS	None			
6	FIND	OIL	PANTRY	EGGS	None			
7	GRAB	OIL	PANTRY	EGGS	OIL			
8	PLACE	EGGS	KITCHEN COUNTER	None	OIL			
9	FIND	BOWL	CABINET	None	OIL			
10	GRAB	BOWL	CABINET	BOWL	OIL			
11	PLACE	BOWL	KITCHEN COUNTER	None	OIL			
12	POUR	OIL	STOVE	None	None			
13	GRAB	EGGS	KITCHEN COUNTER	EGGS	None			
14	CRACK	EGGS	OVER BOWL	None	None			
15	DISCARD	EGG SHELLS	TRASH CAN	None	None			
16	FIND	SALT	PANTRY	None	None			
17	GRAB	SALT	PANTRY	SALT	None			
18	SEASON	EGGS IN BOWL	KITCHEN COUNTER	SALT	None			
19	FIND	PEPPER	PANTRY	None	None			
20	GRAB	PEPPER	PANTRY	PEPPER	None			
21	SEASON	EGGS IN BOWL	KITCHEN COUNTER	PEPPER	None			
22	FIND	KETCHUP	*FRONT OF* *THE REFRIGERATOR*	None	None			
23	GRAB	KETCHUP	*FRONT OF* *THE REFRIGERATOR*	KETCHUP	None			
24	SEASON	EGGS IN BOWL	KITCHEN COUNTER	KETCHUP	None	*10G*		
25	FIND	WHISK	DRAINER BASKET	None	None			
26	GRAB	WHISK	DRAINER BASKET	WHISK	None			
27	BEAT	EGGS IN BOWL	KITCHEN COUNTER	WHISK	None			
28	FIND	PAN	RIGHT OF STOVE	None	None			
29	GRAB	PAN	RIGHT OF STOVE	PAN	None			
30	PLACE	PAN	STOVE	None	None			
31	POUR	BEATEN EGGS	STOVE	None	None			
32	COOK	EGGS IN PAN	STOVE	None	None		MEDIUM	2 MINUTES
33	FIND	SPATULA	DRAINER BASKET	None	None			
34	GRAB	SPATULA	DRAINER BASKET	SPATULA	None			
35	STIR	EGGS IN PAN	STOVE	SPATULA	None			
36	SERVE	SCRAMBLED EGGS	KITCHEN COUNTER	None	None			
37	STANDBY	None	LIVING ROOM	None	None			

The Italic are the added information.

Deletion of information

- The RAP in Table 4 is the result of applying passive modification to the RAP in Table 3 with the modification instruction “Do not use ketchup after all.” A comparison of Table 3 and Table 4 reveals that the process of seasoning with ketchup, which was added earlier as additional information, has been deleted from Table 4 due to passive modification. Specifically, the information shown in bold text in Table 4 has been deleted. This result indicates that a passive modification was applied to delete the information as instructed.

**Table 4:** RAP after passive modification in Task 2 (Deletion of information) in Experiment 2.

	ACTION	OBJECT	ROBOT POSITION	GRIPPER_L	GRIPPER_R	HEAT	DURATION
0	FIND	REFRIGERATOR	KITCHEN	None	None		
1	GRAB	REFRIGERATOR HANDLE	FRONT OF THE REFRIGERATOR	None	REFRIGERATOR HANDLE		
2	OPEN	REFRIGERATOR	FRONT OF THE REFRIGERATOR	None	REFRIGERATOR HANDLE		
3	FIND	EGGS	FRONT OF THE REFRIGERATOR	None	None		
4	GRAB	EGGS	FRONT OF THE REFRIGERATOR	EGGS	None		
5	CLOSE	REFRIGERATOR	FRONT OF THE REFRIGERATOR	EGGS	None		
6	FIND	OIL	PANTRY	EGGS	None		
7	GRAB	OIL	PANTRY	EGGS	OIL		
8	PLACE	EGGS	KITCHEN COUNTER	None	OIL		
9	FIND	BOWL	CABINET	None	OIL		
10	GRAB	BOWL	CABINET	BOWL	OIL		
11	PLACE	BOWL	KITCHEN COUNTER	None	OIL		
12	POUR	OIL	STOVE	None	None		
13	GRAB	EGGS	KITCHEN COUNTER	EGGS	None		
14	CRACK	EGGS	OVER BOWL	None	None		
15	DISCARD	EGG SHELLS	TRASH CAN	None	None		
16	FIND	SALT	PANTRY	None	None		
17	GRAB	SALT	PANTRY	SALT	None		
18	SEASON	EGGS IN BOWL	KITCHEN COUNTER	SALT	None		
19	FIND	PEPPER	PANTRY	None	None		
20	GRAB	PEPPER	PANTRY	PEPPER	None		
21	SEASON	EGGS IN BOWL	KITCHEN COUNTER	PEPPER	None		
	**FIND**	**KETCHUP**	**None**	**None**	**None**		
	**GRAB**	**KETCHUP**	**None**	**KETCHUP**	**None**		
	**SEASON**	**EGGS IN BOWL**	**KITCHEN COUNTER**	**KETCHUP**	**None**		
22	FIND	WHISK	DRAINER BASKET	None	None		
23	GRAB	WHISK	DRAINER BASKET	WHISK	None		
24	BEAT	EGGS IN BOWL	KITCHEN COUNTER	WHISK	None		
25	FIND	PAN	RIGHT OF STOVE	None	None		
26	GRAB	PAN	RIGHT OF STOVE	PAN	None		
27	PLACE	PAN	STOVE	None	None		
28	POUR	BEATEN EGGS	STOVE	None	None		
29	COOK	EGGS IN PAN	STOVE	None	None	MEDIUM	2 MINUTES
30	FIND	SPATULA	DRAINER BASKET	None	None		
31	GRAB	SPATULA	DRAINER BASKET	SPATULA	None		
32	STIR	EGGS IN PAN	STOVE	SPATULA	None		
33	SERVE	SCRAMBLED EGGS	KITCHEN COUNTER	None	None		
34	STANDBY	None	LIVING ROOM	None	None		

Bold indicates information that has been deleted.

Replacement of information

- The RAP in Table 5 is the result of a passive modification to the RAP in Table 4, with the modification instruction “Use butter, not oil, after all.” A comparison of Table 4 and Table 5 shows that the information about oil in 4 is replaced by the information about butter, as in the italic part of Table 5. This result indicates that the passive modification, which replaces information, was applied as indicated by the modification instructions.

**Table 5:** RAP after passive modification in Task 3 (Replacement of information) in Experiment 2.

	ACTION	OBJECT	ROBOT POSITION	GRIPPER_L	GRIPPER_R	HEAT	DURATION
0	FIND	REFRIGERATOR	KITCHEN	None	None		
1	GRAB	REFRIGERATOR HANDLE	FRONT OF THE REFRIGERATOR	None	REFRIGERATOR HANDLE		
2	OPEN	REFRIGERATOR	FRONT OF THE REFRIGERATOR	None	REFRIGERATOR HANDLE		
3	FIND	*BUTTER*	FRONT OF THE REFRIGERATOR	None	None		
4	GRAB	*BUTTER*	FRONT OF THE REFRIGERATOR	*BUTTER*	None		
5	CLOSE	REFRIGERATOR	FRONT OF THE REFRIGERATOR	*BUTTER*	None		
6	FIND	EGGS	FRONT OF THE REFRIGERATOR	*BUTTER*	None		
7	GRAB	EGGS	FRONT OF THE REFRIGERATOR	EGGS	None		
8	PLACE	*BUTTER*	KITCHEN COUNTER	None	None		
9	CLOSE	REFRIGERATOR	FRONT OF THE REFRIGERATOR	EGGS	None		
10	PLACE	EGGS	KITCHEN COUNTER	None	None		
11	FIND	BOWL	CABINET	None	None		
12	GRAB	BOWL	CABINET	BOWL	None		
13	PLACE	BOWL	KITCHEN COUNTER	None	None		
14	GRAB	EGGS	KITCHEN COUNTER	EGGS	None		
15	CRACK	EGGS	OVER BOWL	None	None		
16	DISCARD	EGG SHELLS	TRASH CAN	None	None		
17	FIND	SALT	PANTRY	None	None		
18	GRAB	SALT	PANTRY	SALT	None		
19	SEASON	EGGS IN BOWL	KITCHEN COUNTER	SALT	None		
20	FIND	PEPPER	PANTRY	None	None		
21	GRAB	PEPPER	PANTRY	PEPPER	None		
22	SEASON	EGGS IN BOWL	KITCHEN COUNTER	PEPPER	None		
23	FIND	WHISK	DRAINER BASKET	None	None		
24	GRAB	WHISK	DRAINER BASKET	WHISK	None		
25	BEAT	EGGS IN BOWL	KITCHEN COUNTER	WHISK	None		
26	FIND	PAN	RIGHT OF STOVE	None	None		
27	GRAB	PAN	RIGHT OF STOVE	PAN	None		
28	PLACE	PAN	STOVE	None	None		
29	GRAB	BUTTER	KITCHEN COUNTER	BUTTER	None		
30	PLACE	BUTTER	PAN ON STOVE	None	None		
31	POUR	BEATEN EGGS	STOVE	None	None		
32	COOK	EGGS IN PAN	STOVE	None	None	MEDIUM	2 MINUTES
33	FIND	SPATULA	DRAINER BASKET	None	None		
34	GRAB	SPATULA	DRAINER BASKET	SPATULA	None		
35	STIR	EGGS IN PAN	STOVE	SPATULA	None		
36	SERVE	SCRAMBLED EGGS	KITCHEN COUNTER	None	None		
37	STANDBY	None	LIVING ROOM	None	None		

The italic are the added information.

**Fig. 7:**
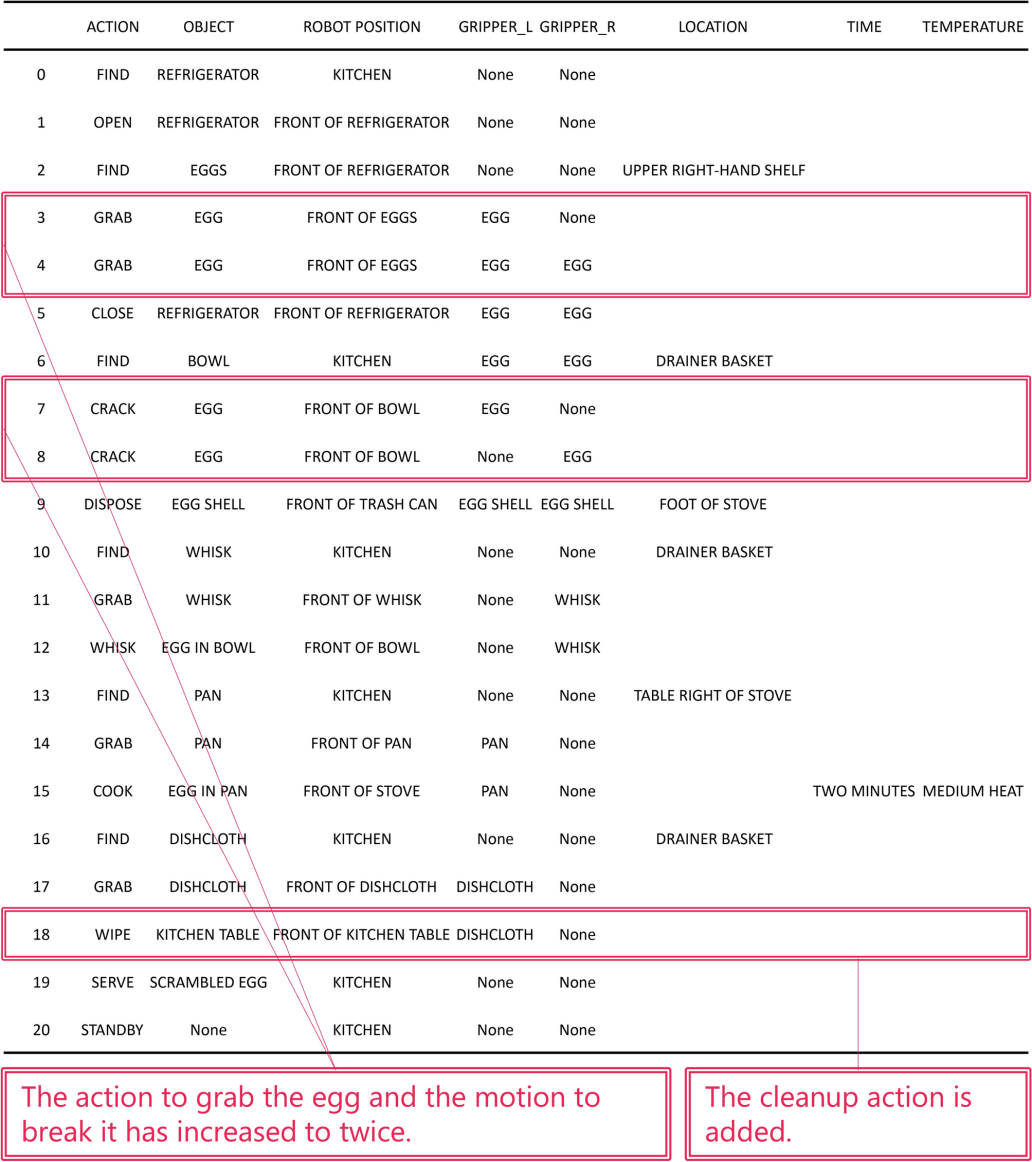
RAP with missing information added by passive modification to Table (b) in Fig. 4, which is the RAP generated by active modification in Task 1 of Experiment 1. Modification Instructions: Please add the following information. Quantity of the eggs is 2. Kitchen table should be wiped down with a dishcloth.

**Fig. 8:**
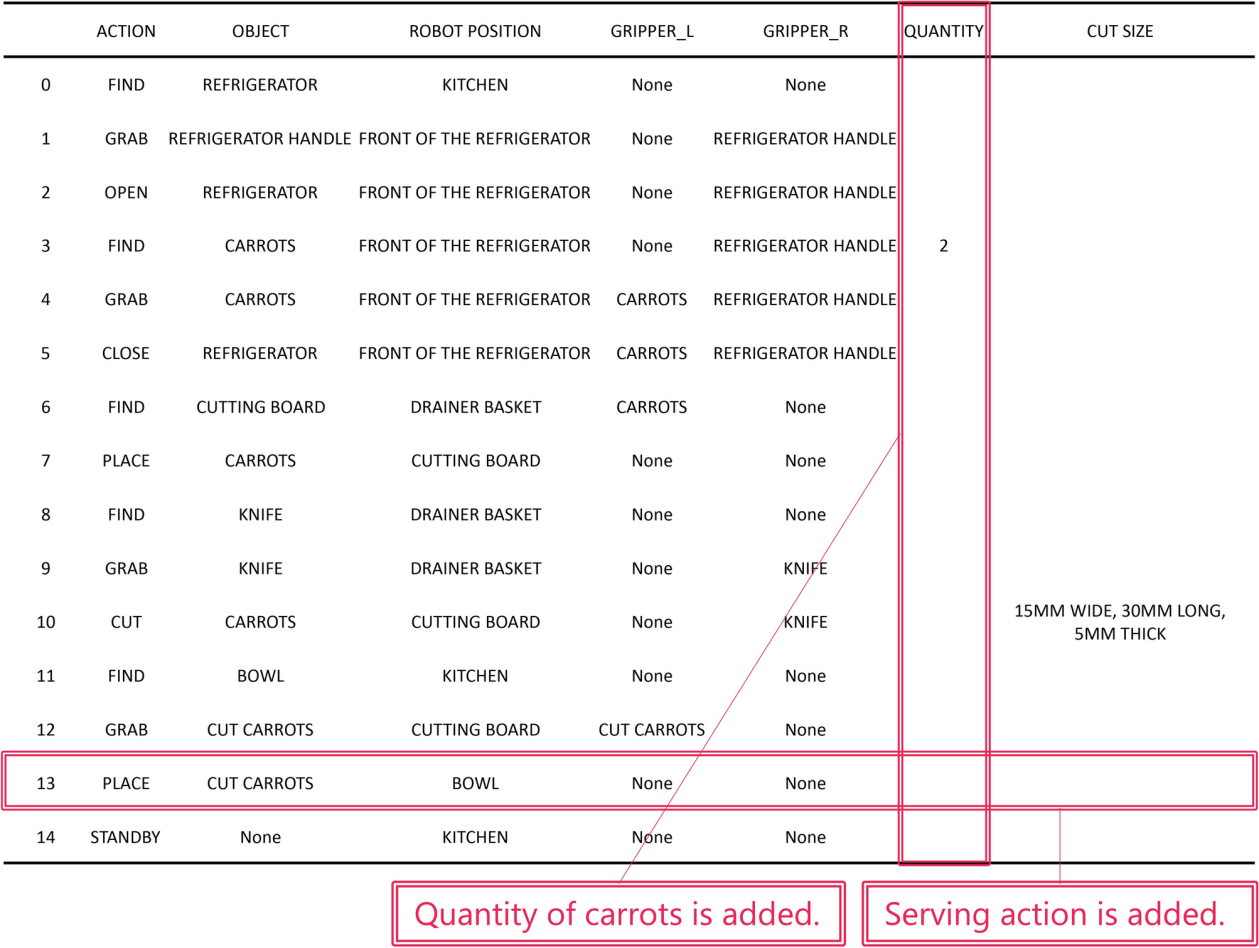
RAP with missing information added by passive modification to Table (b) in Fig. 5, which is the RAP generated by active modification in Task 2 of Experiment 1. Modification Instructions: Please add the following information. Quantity of carrots is 2. Put the cut carrots in a bowl.

Figures 7 and  8 show the results of applying passive modification to the RAPs generated in Experiment 1. Table (b) in Fig. 4, the RAP generated by active modification in Task 1 of Experiment 1, does not include information on quantity of the eggs and cleaning up the mess. These are the pieces of information that should be included to enhance the quality of the scrambled egg cooking task. To compensate for this information, passive modification was applied to the RAP in Table (a) in Fig. 4 using the following modification instructions: “Please add the following information. Quantity of the eggs is 2. Kitchen table should be wiped down with a dishcloth. “ The passive modification using this modification instruction was successful in adding the actions of breaking the second egg and cleaning up the mess, as shown in Fig. 7.

Therefore, the results of Experiment 2 indicate that a combination of active and passive modification is effective. Similarly, Table (b) in Fig. 5, the RAP generated by active modification in Task 2 of Experiment 1, does not include information on the quantity of carrots and food serving. These are the pieces of information that should be included to improve the quality of the carrot-cutting task. These have also been successfully compensated for, as shown in Fig. 8. Therefore, the results of Experiment 2 indicate that a combination of active and passive modification is effective.

### Experiment 3

Finally, we verified the effectiveness of passive modification with visual information in the proposed method. Table 1 shows the RAP before passive modification using visual information in Experiment 3 (Table 1 was also used in Experiment 2-1). Tables 6 and 7 show the results in Experiment 3.

For Task 1 (Addition of location information) of Experiment 3, passive modification that utilized visual information was applied to the RAPs in Table 1 using Fig. 2 and the modification instructions. This passive modification was intended to add location information. The modification instruction was ”Add information on the location of the egg based on the image.” As a result of the experiment, a column named LOCATION and the information on the second shelf from the top (SECOND SHELF FROM TOP) were added, as shown in the italic area of Table 6. This information corresponds to the position of the egg in the Fig. 2.

Similarly, for Task 2 (Addition of feature information) in Experiment 3, passive modification that utilized visual information was applied to the RAPs in Table 1 using Fig. 3 and the modification instructions. Similarly, for Task 2 (Addition of feature information) in Experiment 3, passive modification that utilized visual information was applied to the RAPs in Table 1 using Fig. 3 and the modification instructions. This passive modification is intended to add feature information. The modification instruction was ”Please add information based on the picture about the appearance of the egg.” As a result of the experiment, the column APPEARANCE and the information BROWN SHELL are added, as shown in the italic area of Table 7. This information is consistent with the egg features in Fig. 3. These results show the effectiveness of passive modification using visual information and suggest the scalability of the processing procedure of the proposed method regarding multiple modalities.

**Table 6:** RAP after modifying the output using visual information in Task 1 (Addition of location information) in Experiment 3.

	ACTION	OBJECT	ROBOT POSITION	GRIPPER_L	GRIPPER_R	*LOCATION*	HEAT	DURATION
0	FIND	REFRIGERATOR	KITCHEN	None	None			
1	GRAB	REFRIGERATOR HANDLE	FRONT OF THE REFRIGERATOR	None	REFRIGERATOR HANDLE			
2	OPEN	REFRIGERATOR	FRONT OF THE REFRIGERATOR	None	REFRIGERATOR HANDLE			
3	FIND	EGGS	FRONT OF THE REFRIGERATOR	None	None	*SECOND SHELF* *FROM TOP*		
4	GRAB	EGGS	FRONT OF THE REFRIGERATOR	EGGS	None			
5	CLOSE	REFRIGERATOR	FRONT OF THE REFRIGERATOR	EGGS	None			
6	FIND	OIL	PANTRY	EGGS	None			
7	GRAB	OIL	PANTRY	EGGS	OIL			
8	PLACE	EGGS	KITCHEN COUNTER	None	OIL			
9	FIND	BOWL	CABINET	None	OIL			
10	GRAB	BOWL	CABINET	BOWL	OIL			
11	PLACE	BOWL	KITCHEN COUNTER	None	OIL			
12	POUR	OIL	STOVE	None	None			
13	GRAB	EGGS	KITCHEN COUNTER	EGGS	None			
14	CRACK	EGGS	OVER BOWL	None	None			
15	DISCARD	EGG SHELLS	TRASH CAN	None	None			
16	FIND	SALT	PANTRY	None	None			
17	GRAB	SALT	PANTRY	SALT	None			
18	SEASON	EGGS IN BOWL	KITCHEN COUNTER	SALT	None			
19	FIND	PEPPER	PANTRY	None	None			
20	GRAB	PEPPER	PANTRY	PEPPER	None			
21	SEASON	EGGS IN BOWL	KITCHEN COUNTER	PEPPER	None			
22	FIND	WHISK	DRAINER BASKET	None	None			
23	GRAB	WHISK	DRAINER BASKET	WHISK	None			
24	BEAT	EGGS IN BOWL	KITCHEN COUNTER	WHISK	None			
25	FIND	PAN	RIGHT OF STOVE	None	None			
26	GRAB	PAN	RIGHT OF STOVE	PAN	None			
27	PLACE	PAN	STOVE	None	None			
28	POUR	BEATEN EGGS	STOVE	None	None			
29	COOK	EGGS IN PAN	STOVE	None	None		MEDIUM	2 MINUTES
30	FIND	SPATULA	DRAINER BASKET	None	None			
31	GRAB	SPATULA	DRAINER BASKET	SPATULA	None			
32	STIR	EGGS IN PAN	STOVE	SPATULA	None			
33	SERVE	SCRAMBLED EGGS	KITCHEN COUNTER	None	None			
34	STANDBY	None	LIVING ROOM	None	None			

The italic are the added information.

**Table 7:** RAP after modifying the output using visual information in Task 2 (Addition of feature information) in Experiment 3.

	ACTION	OBJECT	ROBOT POSITION	GRIPPER_L	GRIPPER_R	*APPEARANCE*	LOCATION	HEAT	DURATION
0	FIND	REFRIGERATOR	KITCHEN	None	None				
1	GRAB	REFRIGERATOR HANDLE	FRONT OF THE REFRIGERATOR	None	REFRIGERATOR HANDLE				
2	OPEN	REFRIGERATOR	FRONT OF THE REFRIGERATOR	None	REFRIGERATOR HANDLE				
3	FIND	EGGS	FRONT OF THE REFRIGERATOR	None	None	*BROWN SHELL*	SECOND SHELF FROM TOP		
4	GRAB	EGGS	FRONT OF THE REFRIGERATOR	EGGS	None				
5	CLOSE	REFRIGERATOR	FRONT OF THE REFRIGERATOR	EGGS	None				
6	FIND	OIL	PANTRY	EGGS	None				
7	GRAB	OIL	PANTRY	EGGS	OIL				
8	PLACE	EGGS	KITCHEN COUNTER	None	OIL				
9	FIND	BOWL	CABINET	None	OIL				
10	GRAB	BOWL	CABINET	BOWL	OIL				
11	PLACE	BOWL	KITCHEN COUNTER	None	OIL				
12	POUR	OIL	STOVE	None	None				
13	GRAB	EGGS	KITCHEN COUNTER	EGGS	None				
14	CRACK	EGGS	OVER BOWL	None	None				
15	DISCARD	EGG SHELLS	TRASH CAN	None	None				
16	FIND	SALT	PANTRY	None	None				
17	GRAB	SALT	PANTRY	SALT	None				
18	SEASON	EGGS IN BOWL	KITCHEN COUNTER	SALT	None				
19	FIND	PEPPER	PANTRY	None	None				
20	GRAB	PEPPER	PANTRY	PEPPER	None				
21	SEASON	EGGS IN BOWL	KITCHEN COUNTER	PEPPER	None				
22	FIND	WHISK	DRAINER BASKET	None	None				
23	GRAB	WHISK	DRAINER BASKET	WHISK	None				
24	BEAT	EGGS IN BOWL	KITCHEN COUNTER	WHISK	None				
25	FIND	PAN	RIGHT OF STOVE	None	None				
26	GRAB	PAN	RIGHT OF STOVE	PAN	None				
27	PLACE	PAN	STOVE	None	None				
28	POUR	BEATEN EGGS	STOVE	None	None				
29	COOK	EGGS IN PAN	STOVE	None	None			MEDIUM	2 MINUTES
30	FIND	SPATULA	DRAINER BASKET	None	None				
31	GRAB	SPATULA	DRAINER BASKET	SPATULA	None				
32	STIR	EGGS IN PAN	STOVE	SPATULA	None				
33	SERVE	SCRAMBLED EGGS	KITCHEN COUNTER	None	None				
34	STANDBY	None	LIVING ROOM	None	None				

Italic are the added information.

## Conclusion

This study proposed a method for generating long-horizon offline task planning by questioning and answering with the LLM. The proposed method allows the LLM to collect missing information by analyzing the ambiguity contained in task planning, or to modify the task planning based on human instructions for modification. This method refines the final action plan using one interactive example. The effectiveness of the proposed method was shown through dialog experiments on two types of cooking tasks: cooking scrambled eggs and cutting carrots. Although the dialog experiments presented in this paper are examples of long-horizon robotic tasks, they deal with complex manipulations that imitation learning systems may be able to perform in the future. We believe that the increased amount of information in the RAP provided by the proposed method can serve as an effective guideline for robot motion learning models. Recently developed robot foundation models and imitation learning models use simple task instructions provided via natural language as input and assume motion planning using an LLM. Our proposed method improves the motion planning performance of these robot learning techniques by reducing the cost of motion instruction creation.

However, it was also shown that LLMs have several drawbacks due to the lack of information about the real environment, such as increased token volume, irrelevant questions, and leaps in task planning. Also, since our study targets only long-horizon tasks, it is conceivable that useless questions and answers would increase the planning cost if applied to short tasks. In future studies, we will apply the online planning method using a VLM^6^ to achieve more flexible task planning. In particular, we will attempt to improve the accuracy of each subtask by incorporating success/failure judgments of the robot task using a VLM. In addition, we will attempt to apply our imitation learning model to robot motions^10–14^. Because the speed of motion generation in real robots is an issue affecting the proposed method, we will evaluate the actual planning and task-completion times in future studies, and we will continue to improve the efficiency of the dialogue process.

## Data Availability

The data that support the findings of this study are available from the corresponding author on reasonable request.
